# Biomass fuel use and indoor air pollution in homes in Malawi

**DOI:** 10.1136/oem.2008.045013

**Published:** 2009-08-10

**Authors:** D G Fullerton, S Semple, F Kalambo, A Suseno, R Malamba, G Henderson, J G Ayres, S B Gordon

**Affiliations:** 1 Malawi-Liverpool-Wellcome Clinical Research Laboratories, Universities of Malawi and Liverpool (UK), Blantyre, Malawi; 2 Liverpool School of Tropical Medicine, Liverpool, UK; 3 Scottish Centre for Indoor Air, University of Aberdeen, Aberdeen, UK; 4 Institute of Occupational and Environmental Medicine, University of Birmingham, Birmingham, UK

## Abstract

**Background:**

Air pollution from biomass fuels in Africa is a significant cause of mortality and morbidity both in adults and children. The work describes the nature and quantity of smoke exposure from biomass fuel in Malawian homes.

**Methods:**

Markers of indoor air quality were measured in 62 homes (31 rural and 31 urban) over a typical 24 h period. Four different devices were used (one gravimetric device, two photometric devices and a carbon monoxide (HOBO) monitor. Gravimetric samples were analysed for transition metal content. Data on cooking and lighting fuel type together with information on indicators of socioeconomic status were collected by questionnaire.

**Results:**

Respirable dust levels in both the urban and rural environment were high with the mean (SD) 24 h average levels being 226 μg/m^3^ (206 μg/m^3^). Data from real-time instruments indicated respirable dust concentrations were >250 μg/m^3^ for >1 h per day in 52% of rural homes and 17% of urban homes. Average carbon monoxide levels were significantly higher in urban compared with rural homes (6.14 ppm vs 1.87 ppm; p<0.001). The transition metal content of the smoke was low, with no significant difference found between urban and rural homes.

**Conclusions:**

Indoor air pollution levels in Malawian homes are high. Further investigation is justified because the levels that we have demonstrated are hazardous and are likely to be damaging to health. Interventions should be sought to reduce exposure to concentrations less harmful to health.

Over 2 billion people rely on biomass fuel as their main source of domestic energy. Most of these people live in developing countries such as Malawi, where more than 90% of people cook using biomass fuel (fig 1). Indoor air pollution associated with biomass fuel use is increasingly recognised as a major health concern in the developing world, responsible for an estimated 36% of mortality from respiratory disease as well as contributing to non-respiratory pathology.^1^ ^2^ ^3^ ^4^ ^5^ Pneumonia is the leading cause of death in children <5 years of age and the risk of pneumonia in young children is increased by exposure to unprocessed solid fuels by a factor of 1.8.^6^ ^7^ ^8^ There is an urgent need to describe the smoke exposure that results from biomass fuel use in the developing world, and the burden of disease associated with this exposure.

**Figure 1:**
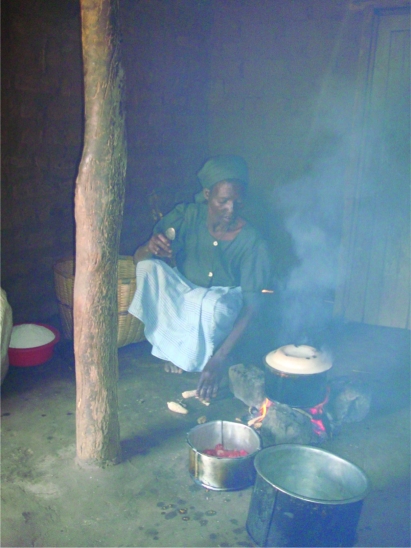
Woman cooking indoors in rural Chikwawa. In the dry season it is more common to cook outdoors or on the veranda (khondi). However, in the wet season or the evenings cooking is often conducted indoors. The situation in the urban environment is similar although indoor cooking (and heating) is more frequent because of cooler average temperatures.

Malawi is located in Southern Africa and has a population of approximately 13 million people; infant mortality is 109/1000.^9^ It is estimated that 95% of people use biomass as their main source of domestic energy.^2^ ^10^ Malawi is typical of many countries in this region of Africa in that it is likely to benefit from appropriate interventions to reduce biomass fuel use and exposure. The World Health Organization (WHO) has produced guidance on acceptable levels of fine particulate matter with an aerodynamic diameter of up to 2.5 μm (PM_2.5_) in inspired air, predominantly based on studies of environmental pollution and outdoor air. These recent guidelines suggest that air quality should be maintained at not higher than a maximum 24 h average concentration of 25 μg/m^3^ PM_2.5_ in order to avoid detrimental health effects. Published studies of biomass burning in homes in Asia have measured PM_2.5_ concentrations over 2000 μg/m^3^ and a small number of studies have reported similar levels in different parts of Africa.^11^ ^12^ ^13^ ^14^ However, there has been no description of biomass fuel use and exposure levels in Malawi.

**What this paper adds**

- Air pollution levels measured in homes in Malawi are high, with 24 h fine particulate matter (PM) levels exceeding the World Health Organization levels for outdoor air in all homes sampled.
- There are significant differences between urban and rural homes. Rural homes have higher PM levels and tend to cook with wood while urban homes have high carbon monoxide concentrations and use charcoal.
- The burden of disease in Malawi that occurs as a result of these exposures is likely to be high and requires both intervention and further study.
- Understanding the concentrations and determinants of exposure to combustion-derived pollution in homes that use biomass fuels is necessary to design, implement and evaluate prevention policies.

The aim of this work was to describe and quantify the concentrations of a variety of markers of indoor air pollution from biomass fuel in Malawian homes. We also aimed to compare four different types of air sampling device in order to assess their suitability for this environment and to help develop appropriate methodology for future exposure assessment work that may be useful in the evaluation of intervention programmes.

## Methods

### Study population and sampling strategy

We sampled urban and rural homes in Southern Malawi. Rural Chikwawa is approximately 30 km from and 1200 m lower altitude than urban Blantyre. The urban homes were randomly selected from 360 Blantyre residents who had previously volunteered for studies at the Malawi-Liverpool-Wellcome Trust Clinical Research Programme (MLW). Selected homes were then visited by a fieldworker, and the residents invited to participate. Rural entomology studies based in Chikwawa had established contacts with the local health surveillance officers in the area. Contact was made with village elders through health surveillance officers and the project was explained to the community. Village homes are similar in design and construction; the first home was chosen as closest in a random direction from the village elder’s house and a snowball sampling strategy was then used to select the other homes. The study was carried out during April 2008 (dry season).

### Questionnaire

Study participants completed an interviewer-administered questionnaire. This was used to obtain information about exposure to potential risk factors, smoking history, occupation and exposure to biomass fuels used in the home for cooking, heating and lighting, details of the house construction, demographic data and an assessment of socioeconomic status.

### Indoor air pollution measurements

A total of 3433 h of data on indoor pollution levels were gathered from 62 homes as detailed in table 1. Samplers were located in the main room of the dwelling and were placed at a height of approximately 1 m from ground level and between 0.5 and 1.0 m from any cooking stove. If more than one device was placed in the same home, they were placed within 10 cm of each other. The main room of the building was defined as the main living area; a room was defined as a separate area divided by a wall that did not necessarily either go to the ceiling or have a door in the entrance to it. Cooking areas were only sampled if they were in the main living areas. Devices were placed in homes between 7:00 and 15:00 in order to capture at least two cooking periods. A second visit was made where possible to extend sampling time by changing batteries if needed.

**Table 1:** Details of homes compared by rural and urban location and air sampling results

Characteristic of household	Urban (n = 31) (%)	Rural (n = 31) (%)	p Value
Female, n (%)	21 (67.7)	26 (83.9)	<0.001
Age (SD)	36.3 (11.1)	43.4 (16.7)	<0.001
Main type of cooking material:			
*Wood*	6 (21)	31 (100)	
*Charcoal*	21 (66)	–	<0.001
*Wood and charcoal*	2 (6)	–	
*Electricity*	3 (9)	–	
Main type of lighting:			
*Simple kerosene lamp, candle and/or flaming torch*	19 (61.3)	27 (86.5)	<0.001
*Hurricane lamp alone*	3 (9.7)	3 (9.7)	NS
*Electricity*	9 (29.0)	1 (3.2)	<0.01
Main cooking location in dry season:			
*Inside*	16 (51.7)	2 (6.4)	
*Separate building*	2 (6.5)	9 (29.0)	<0.01
*Outside*	12 (38.7)	18 (58.1)	
*Inside and outside*	1 (3.2)	1 (3.2)	
*Separate building and outside*	0	1 (3.2)	
Main cooking location in wet season:			
*Inside*	23 (74.2)	7 (22.6)	
*Separate building*	2 (6.5)	12 (38.7)	<0.01
*Inside and outside*	6 (19.3)	12 (38.7)	
Do they heat the home?	16 (51.5)	15 (48.0)	NS
Mean (SD) no of rooms	4.29 (1.4)	4.27 (2.4)	NS
Mean (SD) no of residents	5.03 (1.8)	4.35 (1.7)	<0.001
Mean (SD) no of rooms/residents	0.93 (0.3)	0.99 (0.5)	NS
Mean (SD) sum of household assets	2.29 (1.5)	1.48 (1.1)	<0.001
Number of homes keeping animals	16 (52.0)	27 (87.0)	<0.001
Roof material			
*Grass*	1 (3.2)	21 (67.7)	
*Corrugated material*	29 (93.5)	10 (32.3)	<0.001
*Tiled*	1 (3.2)	0	
Type of window			
*Glass*	7 (22.6)	26 (83.9)	<0.001
*Space only*	24 (77.4)	5 (16.1)	
Total no of air sampling devices used (hours used)			
Gravimetric (apex)			
*Total inhalable dust*	5 (102)	6 (115)	
*Respirable dust*	15 (317)	14 (290)	NS*
Carbon monoxide	30 (671)	28 (569)	<0.001*
SidePak	13 (204)	13 (167)	NS*
UCB	24 (533)	23 (466)	0.001*
Mean (SD) time-weighted average values for each device†			
Gravimetric (mg/m^3^)			
*Total inhalable dust*	0.185 (0.197)	0.268 (0.214)	0.285
*Respirable dust*	0.204 (0.690)	0.811 (0.541)	0.04
Carbon monoxide (ppm)	63.50 (69.86)	16.31 (22.77)	<0.001
SidePak (mg/m^3^)	0.07 (0.08)	0.18 (0.27)	0.343
UCB (mg/m^3^)	0.15 (0.36)	0.25 (0.40)	0.191

Details of homes compared by rural and urban location. Data are from questionnaire and visiting homes and are based on the individual who answered the questionnaire.

*Probability values refer to mean cooking times not total hours of devices were used (see online supplementary table 2).

†All air pollution indices are presented in online supplementary table 2.

UCB, University of California, Berkeley monitor.

#### Gravimetric methods

Airborne particulate mass was measured using standard gravimetric methods.^15^ An apex pump (Casella CEL, Bedford, UK) was attached to either an Institute of Occupational Medicine (IOM) sampling head for total inhalable dust (PM<100 μm) at a flow rate of 2.0 l/min or a Higgins-Dewell cyclone for respirable dust at a flow rate of 2.2 l/min. Both sampling heads contained a pre-weighed 25 mm glass fibre filter with a 0.7 μm pore size.

After sampling, each filter was re-packed and sent back to the UK for re-weighing. Field blanks were used to correct the data for changes in filter weight associated with manipulation. The concentration of PM was calculated by dividing the change in mass of the filter by the volume of air sampled.

#### Photometric methods (SidePak and UCB)

The TSI SidePak AM510 Personal Aerosol Monitor (TSI Incorporated, Shoreview, Minnesota, USA) is a laser photometer (particle size range 0.1–10 μm) which measures aerosol concentrations in the range of 1–20 000 μg/m^3^. Using a Dorr-Oliver cyclone, the device can be used to measure the respirable size fraction of collected PM. In this study, the SidePak was set to log the recorded concentration every minute. Raw data were corrected by applying a calibration factor of 0.295; this was derived from gravimetric data from similar combustion-derived air pollution sources.^16^ ^17^

Our gravimetric and SidePak data are for the respirable size fraction and so are not directly comparable with PM_2.5_. However, particle size distribution of the combustion-derived biomass fuel smoke is likely to be almost entirely less than 2.5 microns in size so we have assumed that respirable dust measurements will closely approximate to PM_2.5_ concentrations.

University of California, Berkeley (UCB) monitors were developed to measure indoor air pollution in the developing world and obtained for this study after personal communication with Professor Kirk Smith (2007) at UCB. The UCB monitor measures PM concentrations of a size fraction similar to respirable dust (0.1–10 μm) using photoelectric methods; it logs changes in concentrations each minute.^18^ Data were downloaded from each device using UCB monitor manager 2.2 software. The UCB is a passive device and therefore uses less battery power allowing it to run for several days, whereas the pumped SidePak instrument runs for approximately 10–15 h.

#### Carbon monoxide (CO) monitors

In settings where there is incomplete combustion of biomass fuels CO levels have been demonstrated to correlate well with airborne PM concentrations.^19^ ^20^ HOBO monitors (Tempcon Instrumentation, Arundel, West Sussex, UK) were used to measure CO concentrations every minute. Data were downloaded from each device using Boxcar 4.3 software.

### Chemical analysis for transition metal content

Filters used in homes were analysed for their metal content using a modification of OSHA ID121 and analysed by inductively coupled plasma/atomic emission spectrometry at the IOM, Edinburgh using standard methods and calibration standards prepared from UKAS-accredited reference material. The samples were digested in concentrated nitric acid on a hotplate at 15°C. Samples were then filtered and made up to 25 ml with distilled H_2_0. The samples were then run in duplicate and a mean of these results was reported.

### Statistical analysis

The clinical database (MS Excel, Microsoft Corp) was verified and then analysed using SPSS V.15. Proportions were compared using the χ^2^ test and air sampling results between rural and urban homes were compared using the non-parametric Mann–Whitney U test. Multiple stepwise linear regression analysis was performed on potential determinants of exposure.

### Ethics

Verbal and written consent to participation in this study was obtained from all volunteers. The study was given ethical approval by the Research Ethics Committee of the College of Medicine, University of Malawi and the Liverpool School of Tropical Medicine.

## Results

### Study population

The characteristics of the urban and rural populations are summarised in table 1. Rural homes cooked exclusively with wood and urban homes used primarily charcoal (p<0.001). In the rural area participants were more likely to cook outside in the dry season while most urban participants cooked indoors. Cooking outside in many cases actually meant cooking on the veranda (khondi); when this was the case much of the smoke was observed entering the main house. During the sampling period two separate cooking locations were often used.

Rural homes in general consisted of one main room divided by partitions that did not go to the ceiling. Most homes, especially in the rural area, did not have a specific kitchen area. The median number of cooking periods captured was two (range 0–4); residents in general burnt biomass fuel between two and three times per 24 h period and there was no difference observed between rural and urban homes in this regard.

Rural homes were more likely to use simple tin lamps fuelled by kerosene (sometimes diesel), or a flaming torch (86%) compared with urban homes (61%) (p<0.01). The rural population had a statistically significantly (p<0.001) lower economic status when assessed using either a simple score of possessions each household owned or the standard of housing (windows and roofing).

### Indoor air pollution measurements

A total of 62 homes (31 urban, 31 rural) were sampled (table 1). High levels of particulate material were measured in both urban and rural samples. A more detailed summary of all the air sampling devices is shown in online supplementary table 2.

#### Gravimetric results

**Figure 2:**
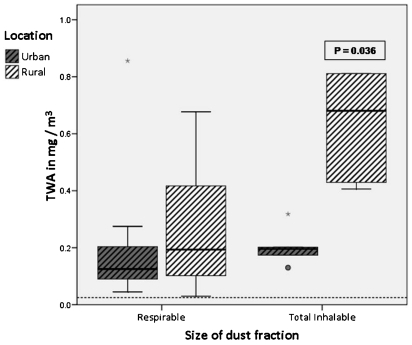
Time-weighted average (TWA) concentrations of respirable and total inhalable particulate matter. There is a significant difference between a rural and urban home in terms of total inhalable dust (p = 0.036) but not for respirable dust. The dotted line represents the World Health Organization outdoor air quality level for particulate matter with an aerodynamic diameter of up to 2.5 μm (25 μg/m^3^). It has been added to highlight that all the homes sampled were above this level, and 80% were over four times greater. An outlier home, with a TWA of total inhalable dust of 1860 μg/m^3^, has been removed for clarity. If this home was excluded from the analysis the statistical difference between the urban and rural population increased to p = 0.008.

Forty homes were sampled (20 rural, 20 urban) ranging from 8.73 h to 25.7 h in duration, mean (SD) 20.6 h (3.07 h). Twenty-nine homes were sampled using respirable dust sampling heads (range 30–856 μg/m^3^; mean (SD) 226 μg/m^3^ (206 μg/m^3^)); 11 homes were sampled using total inhalable dust sampling heads (range 130–1860 μg/m^3^; mean (SD) 535 μg/m^3^ (499 μg/m^3^)). In summary, the total inhalable dust levels were significantly higher in the rural compared with the urban environment (p<0.01). The time-weighted average (TWA) levels for respirable dust were comparable between rural and urban locations (fig 2). Multiple linear regression analysis implied that higher total inhalable dust levels were associated with cooking with wood (p = 0.036, beta  =  0.065) and that higher respirable dust levels were potentially associated with more polluting lighting sources (p = 0.046, beta  =  0.403).

#### Photometric results

##### SidePak aerosol monitor

**Figure 3:**
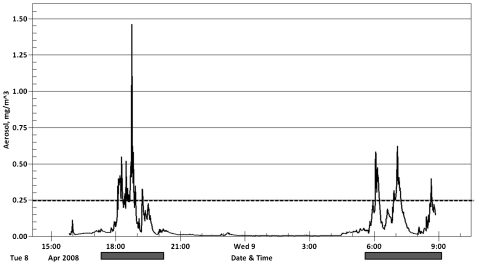
A plot of particulate matter against time, obtained using the photometric (SidePak) device. Cooking periods are highlighted with the grey boxes on the x axis. In this particular home cooking took place on the veranda and the device was placed inside the main room of the home (approximately 1.5 m from the fire). Peaks associated with cooking are seen and for over 1.5 h of the day levels of particulate matter with an aerodynamic diameter of up to 2.5 μm are >250 μg/m^3^, a level deemed hazardous by the US Environmental Protection Agency.

Twenty-six homes were sampled (13 urban, 13 rural). The duration of sampling ranged from 3.43 to 24.6 h, mean (SD) 14.32 h (4.98 h). The mean TWA respirable dust concentration across all homes was 120 μg/m^3^ (range 7–891 μg/m^3^, SD 199 μg/m^3^). There was no significant difference between the levels measured in the rural and urban environments (p = 0.34). The median peak values for the rural and the urban environments was 950 μg/m^3^. The maximum value that was recordable using a SidePak was 5900 μg/m^3^; a typical home is represented in fig 3.

##### UCB measurement

Forty-seven homes (24 urban, 23 rural) were sampled. The duration of sampling was 17.4–25.1 h, mean (SD) 21.3 h (2.10 h). The mean TWA for all homes was 204 μg/m^3^ (range 1–1900 μg/m^3^, median 80 μg/m^3^ and SD 381 μg/m^3^). The TWA differences between the rural and urban environment were not significant. The duration when PM concentrations in homes exceeded 250 μg/m^3^ was higher in rural homes than in urban homes; 52% of rural houses spent >1 h per day above the 250 μg/m^3^ concentration compared with only 17% of urban homes (p = 0.112). The median peak values for the rural and the urban environments was 1400 μg/m^3^.

#### CO measurement

Fifty-eight homes (30 urban, 28 rural) were sampled. The mean (SD) duration of sampling was 21.4 h (2.20 h); range (17.4–26.8 h). The mean CO TWA for all homes was 4.08 ppm (range 0.2–24.6; median 1.80 ppm; SD 5.36).There was a significantly higher level of CO in urban homes compared with rural homes (6.14 ppm vs 1.87 ppm; p<0.001). The same was found when peak values and time above 5 ppm were analysed. Multiple linear regression analysis associated CO levels with the use of charcoal (p<0.001, beta 0.539).

### Transition metal content

Twenty filters (10 rural, 10 urban) were analysed for aluminium, arsenic, cobalt, chromium, copper, mercury, nickel, selenium and vanadium. Levels of arsenic, cobalt, mercury, nickel, selenium and vanadium collected on the glass fibre filters were <0.1 μg (the limits of detection of the assay); aluminium, chromium and copper were detected in small quantities. The mean metal weight on the filter for all homes was aluminium  =  13.40 μg; chromium  =  0.21 μg; copper  =  0.48 μg. The percentage mass values of the filters were aluminium  =  6.25; chromium  =  0.037; copper  =  0.11. There was no significant difference in transition metal level content when compared by fuel type (wood and charcoal) in this study.

## Discussion

This is the first assessment of indoor air quality in Malawi. Concentrations of respirable dust in this sample were high and exceeded WHO recommended levels of 25 μg/m^3^ in all homes sampled. In 80% of homes, the PM levels measured were four times greater than the WHO level for outdoor air quality.

Respirable dust levels were similar in both rural and urban homes. This is different to results from other developing world countries where rural levels tend to be significantly higher than those measured in urban households.^12^ Total inhalable dust levels were higher in the rural environment. This may be explained by wood use, as implied by the multiple linear regression analysis, but also by the greater prevalence of domestic animals kept for food, or the rural environment being significantly dustier and homes being of more simple construction. Homes in the urban environment experienced significantly higher levels of CO. This is almost certainly due to charcoal burning and homes being less well ventilated perhaps due to better construction than homes in the rural location. CO concentrations of the levels measured in this study were high and similar levels have been seen in other studies where associations with health effects have been seen (although in these studies CO is probably acting as a surrogate for other pollutants).^21^ ^22^ ^23^ Whilst fine PM levels were not significantly different between the rural (wood burning) and urban (charcoal) homes in our study, there was a trend to higher levels in the rural environment and data from Mozambique suggest that homes that burn charcoal produce less PM pollution.^24^ Data from neighbouring countries to Malawi are limited; however, similar results in single studies have been seen in Zimbabwe, Tanzania, Kenya and Mozambique.^14^ ^24^ ^25^ ^26^

The peaks of PM exposure reached ⩾30 000 μg/m^3^ using the UCB-PM monitor and for CO levels were >300 ppm. The environmental standard used to quantify air pollution is the TWA. This measure has been used in our study in order to allow comparison with published data but it may underestimate the health impact of high peak levels to which individuals were exposed. We suggest that the duration during which individuals are exposed to fine PM >250 μg/m^3^ may be a better predictor of long-term lung damage. The length of time that indoor air concentrations of fine PM exceeded the US Environmental Protection Agency (PM_2.5_) “hazardous” 24 h mean level of 250 μg/m^3^ in rural homes was typically 1–2 h per day. Whether duration above a certain threshold (eg, 250 μg/m^3^) is more strongly associated with health effect than TWA levels needs to be tested by epidemiological work and in vitro toxicological studies.

The data reported here were obtained using static monitors within participants’ homes. Data from occupational settings comparing personal and static sampling indicate that personal sampling generally reveals higher exposure concentrations than those estimated with static monitors as workers often interact closely with sources.^27^ ^28^ Women who are involved in cooking and fire-tending activities that involve close contact with the stove or fire may have higher peak exposures than those reported here. This effect may be hard to detect, however, as in pilot studies we found that individuals wearing monitors were clearly identifiable in the community which in turn led to some criticism by neighbours and may have led them to modify their usual cooking behaviours on the day of sampling. This study has not addressed the subject of seasonal variation in exposure. There are three distinct seasons in Malawi (wet, dry, and cold) that alter cooking and heating practices; we sampled during the dry season. The questionnaire data (table 1) showed that there is an increase in indoor cooking during the wet season and so there is likely to be a subsequent increase in exposure levels, which was not accounted for in this work.

Transition metal content of biomass fuel smoke derived from charcoal and wood in this region of Malawi contains much lower levels of redox active fine particulates than levels reported elsewhere. The transition metal content of PM potentially could cause inflammation and damage DNA.^29^ ^30^ A biological hierarchy seems to exist in terms of tissue damage, with low-valence transition metals being key to PM bioreactivity.^31^ In India, where individuals burn dung cake, PM contains much higher levels of transition metals, including arsenic which has been shown to have a biological effect.^32^ ^33^ Levels reported from urban airborne PM in Mexico contained transition metal concentrations of 0.03–1000 ng/m^3^ and it is possible that the sensitivity of the assay used in this study requires larger quantities of PM to be collected and examined before definitive statements can be made about the constituents, and relative toxicology of biomass fuel smoke from Malawi.

Evidence of varying degrees of certainty exists that biomass fuel smoke is associated with acute lower respiratory tract infections and low birth weight in children,^34^ ^35^ lung cancer, chronic obstructive pulmonary disease, interstitial lung disease, tuberculosis, cardiovascular disease and cataracts in adults.^4^ ^36^ ^37^ ^38^ ^39^ ^40^ ^41^ ^42^ ^43^ However, despite indoor air pollution from biomass fuel potentially affecting a larger number of individuals globally there are less data on biomass smoke specific exposures, both in terms of levels of exposure and health effects.^44^ Data from Kenya have explored the exposure–response relationship of biomass fuel and lower respiratory tract infections^13^; this has suggested that the benefits of reduced exposure to PM_10_ may be larger for average exposure less than about 1000–2000 μg/m^3^, but to date there are no further data on this from Africa. Recent epidemiological data from outdoor air in the USA have shown that a decrease of 10 μg/m^3^ in the concentration of fine PM was associated with an estimated increase in mean (SE) life expectancy of 0.61 (0.20) years.^45^ The effect of air pollution indoors has been investigated more in the context of second hand tobacco smoke, nevertheless reductions in smoke levels have led to a reduction in both mortality and morbidity.^46^ ^47^ If appropriate interventions were instituted in countries such as Malawi, with subsequent substantial reductions in exposures to PM and other constituents of biomass, the health benefits are likely to be large. Potential immediate interventions could include simple education strategies, changes to homes to improve ventilation or alternatively more efficient stoves (eg, rocket stoves; www.aprovecho.org) that burn the same fuel.

In conclusion, levels of indoor air pollution in rural and urban Malawian homes were sufficiently high to be a cause for concern. Our data will hopefully prove to be important as a baseline for any proposed intervention study aimed at reducing exposure levels in Malawi and other similar countries.
